# Antibody Modified Gold Electrode as an Impedimetric Biosensor for the Detection of *Streptococcus pyogenes*

**DOI:** 10.3390/s20185324

**Published:** 2020-09-17

**Authors:** Natalia Malinowska, Wioleta Białobrzeska, Tomasz Łęga, Katarzyna Pałka, Karolina Dziąbowska, Sabina Żołędowska, Elżbieta Czaczyk, Katarzyna Pala, Dawid Nidzworski

**Affiliations:** 1 SensDx, 14b Postępu St., 02-676 Warszawa, Poland; wioletta.bialobrzeska@etongroup.eu (W.B.); tomasz.lega@etongroup.eu (T.Ł.); katarzyna.palka@etongroup.eu (K.P.); karolina.dziabowska@etongroup.eu (K.D.); ela@etongroup.eu (E.C.); katarzyna.pala@etongroup.eu (K.P.); dawid@etongroup.eu (D.N.); 2 Institute of Biotechnology and Molecular Medicine, 3 Trzy Lipy St., 80-172 Gdańsk, Poland; s.zoledowska@ibmm.pl

**Keywords:** biosensor, 4-aminothiophenol, gold electrode, impedance spectroscopy, antibodies, *Streptococcus pyogenes*

## Abstract

*Streptococcus pyogenes* is a known cause of a wide spectrum of diseases, from mild and acute to severe invasive infections. This paper concerns the development of a novel impedimetric biosensor for the detection of the mentioned human pathogen. The proposed biosensor is a gold disk electrode modified with commercially available antibodies attached to the surface of the electrode by carbodiimide chemistry. The conducted tests confirmed the specificity of the antibodies used, which was also demonstrated by the results obtained during the detection of *S. pyogenes* using electrochemical impedance spectroscopy. The developed sensor successfully detected the presence of *S. pyogenes* in the sample and the detection limit was calculated as 9.3 cfu/mL. The results obtained show a wide linear range for verified concentrations of this pathogen in a sample from 4.2 × 10^2^ to 4.2 × 10^6^ cfu/mL. Furthermore, the optimal experimentally determined time required to perform pathogen detection in the sample was estimated as 3 min, and the test did not lead to the degradation of the sample.

## 1. Introduction

*Streptococcus pyogenes*, called Group A *Streptococcus* (GAS), is widely occurring and one of the most frequent, exclusive to humans pathogens. This gram-positive microbe is known as a cause of a broad spectrum of diseases [1,2,3,4,5,6,7]. Exemplary are mild acute infections such as pharyngitis or tonsil inflammation, skin infections (impetigo, pyoderma, erysipelas, or cellulitis), or severe invasive infections, such as endocarditis, bacteraemia, puerperal fever, scarlet fever, or necrotising fasciitis [8,9,10,11,12]. Nowadays, GAS remains a major health concern because of rapidly progressive diseases and also due to severe after-effects of untreated infections [13,14,15].

High incidence and severity of GAS pathogens occur due to productions of a large number of virulence factors, including surface proteins (such as M proteins, protein F), hyaluronic acid capsules, or secreted enzymes and toxins [2]. The surface of *S. pyogenes* is extraordinarily complex and is composed of capsular polysaccharide, cell wall, lipoteichoic acid, and proteins [16]. Commercially available assays can recognize the surface proteins of *S. pyogenes* [17].

Nowadays, numerous advanced techniques can be involved in GAS recognition and identification [18,19,20,21]. Methods called nucleic acid amplification testing (NAAT) such as LightCycler Strep A assay combine PCR reaction and real-time detection of an amplified product; the Cobas Liat Strep A based on nucleic acid purification and detection also through PCR technique, are used in clinical routine [22,23,24,25,26]. Comparing to conventional detection methods, NAAT provides auspicious results with sensitivity and specificity reaching 97% and 93%, respectively (in Liat Strep A technique), or 93% and 98%, respectively (in LightCycler Strep A assay) in a relatively short time (from 15 to 60 min). Moreover, the NAAT techniques have received Food and Drug Administration (FDA) clearance [13,22,23].

Electrochemical detection is another promising diagnostic method. Sensors capable of recognition of specific bacteria can base on protein and DNA detection as well on immunoassays [27,28,29,30,31,32,33,34,35,36]. Ahmed et al. [37] proposed an electrochemical sensor for the *Streptococcus pyogenes* detection from human saliva. The authors suggested the modification process through polytyramine film immobilized with the biotin-NeutrAvidin complex. This approach requires previous antibodies biotinylation; however, this method demands a relatively long time of operation and specific sample preparation.

Here we develop a novel sensitive, rapid electrochemical immunosensor based on impedance measurements. This type of sensor for the detection of *Streptococcus pyogenes* has never been published in the literature before. The entire surface modification process has been developed in a way that guarantees high sensitivity of the sensor and eliminates the problem of sample decomposition during the test. The surfaces of gold disk electrodes were easily modified in a three-step procedure. Commercially available antibodies were anchored using carbodiimide chemistry on the surface of the electrodes on which a self-assembled layer had been previously formed with 4-aminothiophenol. The described sensor shows very good repeatability of measurements, satisfactory sensitivity, and specificity. In the future, our sensor can serve as a tool for point-of-care diagnostics after miniaturizing this system.

## 2. Materials and Methods

### 2.1. Materials

Phosphate buffered saline (1 × PBS, pH 7.4), bovine serum albumin (BSA), and glutaraldehyde solution (GA, 25%) were purchased from Sigma-Aldrich (St. Louis, MO, USA). 4-aminothiophenol (4-ATP, 96%) and *Streptococcus pyogenes* Group A Polyclonal Antibody (anti-Spy) were obtained from Thermo Fisher Scientific (USA). Pure ethanol, potassium chloride, K_3_[Fe(CN)_6_], and K_4_[Fe(CN)_6_] × 3H_2_O were acquired from Chempur (Poland). Artificial saliva was provided by Pickering Laboratories (USA). 0.1% BSA was prepared in 10 mM, pH 7.4 sterile phosphate buffer. All aqueous solutions were prepared using ultrapure water (HydroLab).

All electrochemical measurements were carried out using a potentiostat-galvanostat system (Metrohm, Autolab, The Netherlands) in a standard three-electrode assembly in a Faraday cage (Lambda System, Poland). Gold disc electrodes (diameter: 1.6 mm, surface area: ca. 0.02 cm^2^), were obtained from Mineral (Poland) and utilized as the working electrode, while Ag/AgCl/0.1 M NaCl (Mineral, Poland) functioned as the reference electrode and Pt sheet (Mennica-Metale, Poland) as the counter electrode. All electrochemical experiments were carried out in 3 mL of 5 mM K_3_[Fe(CN)_6_]/K_4_[Fe(CN)_6_] redox system containing 0.1 M KCl at room temperature. The experimental conditions of cyclic voltammetry (CV) were a potential range from −0.15 to 0.40 V and a scan rate of 100 mV/s. Electrochemical impedance spectroscopy (EIS) was recorded at the formal potential of the redox couple (0.16 V), in the frequency range between 10 kHz and 1 Hz. All measurements were repeated on three separate electrodes to obtain repeatability of measurements and thus, reliability of the biosensor.

*Streptococcus pyogenes* ATCC 700294, *Acinetobacter baumannii* ATCC 19606, and *Haemophilus influenzae* ATCC 51907 were purchased from ATCC (US). *Streptococcus pyogenes 2318, Streptococcus pyogenes 917*, *Streptococcus pyogenes 915*, *Streptococcus pyogenes 2317*, *Klebsiella pneumoniae*, and *Staphylococcus aureus MRSA* were obtained from PCM (Poland). All strains were grown overnight at 37 °C with shaking (200 rpm) in BHI (Brain Heart Infusion Broth, Sigma-Aldrich). In the case of *H. influenzae*, media were supplemented with β-NAD^+^ and heme-histidine (sBHI) as described elsewhere [38]. 1 mL samples of overnight cultures were centrifuged and resuspended in PBS to bring the optical density (OD_600_) to 1.0. Serial dilutions in PBS were prepared (10^−2^, 10^−3^, 10^−4^, 10^−5^, 10^−6^). Each dilution was plated on BHI plate (or sBHI for *H. influenzae*) and incubated overnight at 37 °C. The number of bacteria for each dilution was counted, the results were averaged, and the cfu/mL was calculated as follows (1):

cfu/mL = (number of colonies × dilution factor)/(volume of culture plate).(1)

Afterward, serial dilutions in PBS for electrochemical measurements were prepared (4.2 × 10^2^, 4.2 × 10^3^, 4.2 × 10^4^, 4.2 × 10^5^, 4.2 × 10^6^ cfu/mL).

### 2.2. Preparation of the Biosensor

Before use, the gold electrodes were mechanically polished with 1 µm and 0.04 µm alumina slurry followed by washing with ultrapure water. The electrodes were dipped in the absolute ethanol and sonicated for 3 min to remove alumina residues. Afterward, the gold electrodes were rinsed with ultrapure water and dried in a stream of pure argon.

**Figure 1:**
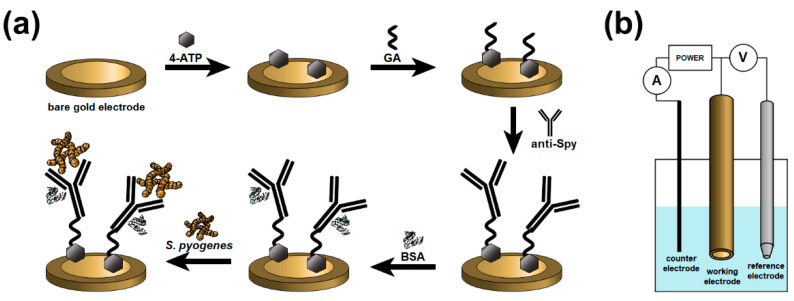
(**a**) Schematic diagram of the *Streptococcus pyogenes* modification method and detection process. (**b**) Experimental setup of the sensor measurements.

Immediately after the cleaning procedure, the gold electrodes were flushed with absolute ethanol, dried with an argon stream, and immersed into 0.1 M ethanolic solution of4-aminothiophenol (4-ATP) for 19 h at 4 °C. For the next step, the electrodes were rinsed with pure ethanol and gently dried with an argon stream. Subsequently, the electrodes were dipped into the 2.5% aqueous solution of glutaraldehyde (GA) and placed for 15 min in a dark place. Next, the electrodes were rinsed with ultrapure water and dried in a stream of argon. Later, the electrodes were left to incubate for 90 min with 4 µL of 31 µg/mL anti-Spy diluted in 1 × PBS. After that, the electrodes were washed with phosphate buffer, and 4 µL of 0.1% bovine serum albumin solution (BSA) was dropped onto their surface. Then, the electrodes were incubated for 10 min at 4 °C. Finally, the gold electrodes were washed with phosphate buffer, ultrapure water, and gently dried in a stream of pure argon. The schematic representation of the modification and detection process is given in Figure 1a. The experimental setup is presented in Figure 1b.

## 3. Results

### 3.1. Anti-Spy Immobilization on the Surface of the Electrodes

**Figure 2:**
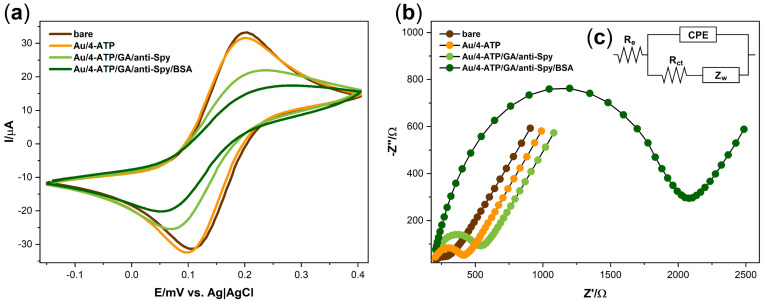
(**a**) Cyclic voltammograms of 5 mM K_3_[Fe(CN)_6_]/K_4_[Fe(CN)_6_]/0.1 M KCl on the bare gold electrode and after modification steps at a scan rate of 100 mV/s; (**b**) Impedance spectra for 5 mM K_3_[Fe(CN)_6_]/K_4_[Fe(CN)_6_]/0.1 M KCl registered at the formal potential of the redox couple [Fe(CN)_6_]^3-/4-^ on the bare gold electrode and after modification steps; (**c**) the equivalent electric circuit (EEQC) model applied to fit the impedance measurements.

The cyclic voltammetry and electrochemical impedance spectroscopy measurements were performed to investigate the correctness of antibodies anchoring on the electrode surface (Figure 2). An amount of 5 mM K_3_[Fe(CN)_6_]/K_4_[Fe(CN)_6_] in 0.1 M KCl was chosen for the characterization of the modified surface.

Cyclic voltammetry measurements were performed in the range from −0.15 to 0.40 V (scan rate of 100 mV/s) to investigate the electrochemical behavior of the mentioned redox couple (Figure 2a). At the bare electrode, the oxidation (E_OX_) and reduction potentials (E_RED_) were found at 202 mV and 108 mV, respectively, with the peak-to-peak separation of 94 mV (ΔE) (Table 1). With each subsequent modification stage, the difference between the potentials’ values increases, and the current value decreases. It can be seen that the electrode capacity is reduced during the modification process, which indicates that electron transfer through the electrode surface is hampered. More visible changes occur after the immobilization of anti-Spy antibodies, which confirm their attachment to the surface of the electrode. After incubating the electrode with BSA, in order to block the free surface, the oxidation and reduction peaks cannot be unambiguously identified. This phenomenon shows that the electron transfer between the redox system and the electrode is blocked.

Figure 2b presents the impedance spectra of the bare and modified electrode recorded in 5 mM K_3_[Fe(CN)_6_]/K_4_[Fe(CN)_6_] in 0.1 M KCl at the formal potential of redox couple (0.16 V). The EIS measurements were conducted in a frequency range between 10 kHz and 1 Hz. All electrochemical impedance spectra were analyzed using an equivalent electric circuit (EEQC) R_e_[CPE(R_ct_W)] which includes electrolyte resistance (R_e_), constant phase element (CPE), charge transfer resistance (R_ct_), and Warburg element (W) for diffusional resistance (Figure 2c). The results obtained using this method are given in Table 1. The comparison of modification levels can be made using the R_ct_ parameter. It can be seen that the charge transfer resistance value increases from 190 Ω to 256 Ω after surface modification with 4-aminothiophenol, which indicates the formation of a self-assembled monolayer on the surface of the bare electrode. Further increase of this parameter (up to 446 Ω) corresponds to the antibodies anchor on the surface. The highest value of 1790 Ω is the result of using a BSA solution, which effectively blocked empty spots on the electrode surface. Such a gradual increase in the value of this parameter, along with the subsequent stages of modification, indicates the hindering of electron transfer from the electrolyte to the biolayer due to the increase in its thickness.

As expected, the results obtained during the modification process showed agreement between electrochemical impedance spectra and cyclic voltammograms, which indicates that the surface of the electrodes has been successfully modified.

### 3.2. Electrochemical Detection of Streptococcus Pyogenes

**Figure 3:**
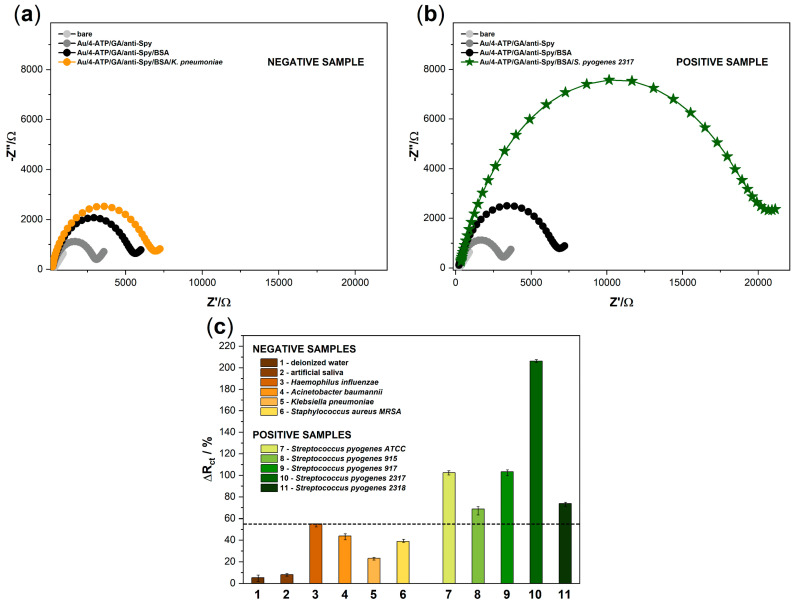
The impedance spectra registered for (**a**) negative sample containing *K. pneumoniae* and (**b**) positive sample containing *S. pyogenes 2317*. The scale in figures (**a**) and (**b**) has been unified to show differences in the impedance values obtained for negative and positive samples; (**c**) the plot of R_ct_ percentage changes as a response of the biosensor after incubation with negative and positive samples. The error bars show the standard deviation for three individual experiments. The concentration of pathogens was kept at 10^6^ cfu/mL.

In order to demonstrate the specificity of the developed biosensor, a series of measurements with negative samples was planned. Four different pathogens were selected (*Haemophilus influenzae, Acinetobacter baumannii, Klebsiella pneumoniae,* and *Staphylococcus aureus MRSA*) (Figure 3). We also used deionized water and commercially available artificial saliva (which are the components of the potential human swab) to exclude possible cross-reactions and prove the correctness of further assumed studies; verification of the sensor on biological samples, which are planned but yet to be done will be further published in the next paper. Figure 3a presents the spectra obtained for a sample containing *Klebsiella pneumoniae* as an example of the results obtained for negative samples. Five different strains of *Streptococcus pyogenes* were used as positive samples to demonstrate the specificity of the anti-*Streptococcus pyogenes* antibodies. Exemplary spectra obtained for a sample containing *Streptococcus pyogenes 2317* as one of the positive samples are shown in Figure 3b. Figure 3c shows that the plot of charge transfer resistance parameter changes as a response of the biosensor after incubation with negative and positive samples, which were obtained using the EEQC model. The biosensor response was determined by the difference in R_ct_ values before and after adding the sample onto the biosensor’s surface and calculated from the following Equation (2):

ΔR_ct_ = (R_ct_^sample^ − R_ct_^sensor^)/(R_ct_^sensor^), (2)

Each of the negative samples was tested on a separate electrode, but their mixtures were also investigated.

According to the assumptions, there was no significant increase in the value of the R_ct_ parameter for the first two tested samples: deionized water, artificial saliva. Additionally, these changes did not exceed 10% (Figure 3). Negative samples containing pathogens: *Haemophilus influenzae*, *Acinetobacter baumannii*, *Klebsiella pneumoniae,* and *Staphylococcus aureus MRSA* reached values below 54%. These values may be the result of physical clogging of the electrode surface, and the reason may be a high concentration of bacteria in the sample. The highest value obtained for the negative sample was considered as the limit value separating the positive and negative results. The percentage of changes in the charge transfer resistance parameter for positive samples was more significant and ranged from 68.73 to 206.28%. We conclude that the anti-Spy antibodies are highly specific to target bacteria as they properly bind to five different *S. pyogenes* strains used for the experiment. All measurements were repeated on a series of three electrodes to confirm the lack of influence of negative samples on further measurements. Furthermore, the relative standard deviations (RSD) took values from 2.3 to 7.1%, which indicates the high stability of the proposed system.

**Figure 4:**
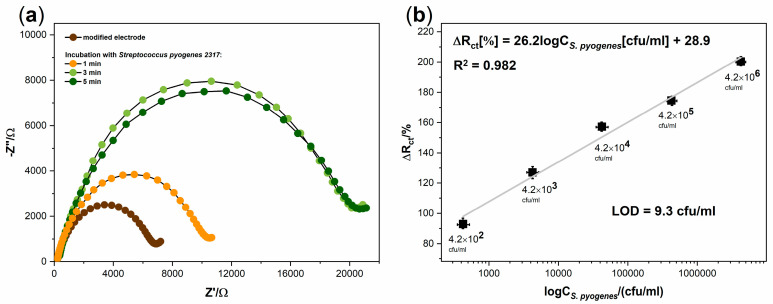
(**a**) the impedance spectra results for the detection of *Streptococcus pyogenes 2317* in time (1, 3, 5 min) at concentration 10^6^ cfu/mL; (**b**) the linear plot of the percentage changes in charge transfer resistance vs. decimal logarithm of the concentration of *Streptococcus pyogenes 2317*.

The next stage of our work was to examine the effect of incubation time of the modified electrode with the positive sample. Figure 4a shows the electrode responses in the form of EIS spectra for three different incubation times of the electrode with a positive sample at room temperature. EIS measurements were carried out after 1, 3, and 5 min of the mentioned incubation. It can be seen that the spectra after 3 min were practically unchanged. This phenomenon indicates that the maximum possible amount of antigen has been anchored on the surface of the biosensor. Moreover, for negative samples, the EIS spectrum was also stable after 3 min (data not shown). This observation allows us to determine this time as optimal for further measurements.

The final stage of the research was to determine the performance of the developed biosensor. For this purpose, a series of electrodes were prepared by modifying their surfaces accordingly to the proposed procedure. Each electrode was incubated for 3 min with a different bacterial concentration of *Streptococcus pyogenes 2317* in 1 mL of the sample. Five concentrations ranging from 4.2 × 10^2^ to 4.2 × 10^6^ cfu/mL were tested. All spectra were registered in a 5 mM K_3_[Fe(CN)_6_]/K_4_[Fe(CN)_6_] containing 0.1 M KCl solution. The value of the R_ct_ parameter was determined using EEQC fitting for each obtained spectra. The increase in the R_ct_ value after the bacterial binding was calculated. Figure 4b shows the plot of the changes in charge transfer resistance vs. decimal logarithm of the concentration of *Streptococcus pyogenes 2317*. For the lowest pathogen concentration (4.2 × 10^2^ cfu/mL) in the sample, the percentage change in the R_ct_ parameter value was 92.89%. It is 1.68 times higher than the limit value for negative samples; therefore, it still allows the sample to be identified as positive. The value of this parameter was 200.32% for the sample with the highest pathogen concentration (4.2 × 10^6^ cfu/mL). The impedimetric sensor showed a wide linear range for all concentrations tested. The linear regression was determined and presented in the decimal logarithm of the *S. pyogenes 2317* concentration. The linear regression equation can be expressed as ΔR_ct_[%] = 26.2logC_S.pyogenes_[cfu/mL] + 28.9 with the correlation coefficient of R^2^ = 0.982. Additionally, the values of the RSD were calculated, which ranged from 3.1 to 6.7%. The calculated limit of detection (LOD) was found to be 9.3 cfu/mL (S/N = 3) and the received LOD value and linearity ranges of this assay were compared with other electrochemical methods for *S. pyogenes* detection and presented in Table 2.

## 4. Discussion

A novel diagnostic method for the detection of human pathogens should focus mainly on high specificity, selectivity, and relatively short time of detection to allow general practitioners to react immediately. The sensor we propose works on the basis of electrochemical impedance spectroscopy, which allows quick identification of the samples. The presence of *Streptococcus pyogenes* in the sample is being confirmed within 3 min. The limit of the detection value for the designed sensor was set to 9.3 cfu/mL. The obtained results are linear in the whole range of tested concentrations from 4.2 × 10^2^ to 4.2 × 10^6^ cfu/mL, and the R^2^ value is equal to 0.982, which indicates good sensor performance. Further research will cover the scope of measurements using samples coming from a patient that will be collected in the form of a throat swab. The results are planned to be presented in the next publication. In addition, measurements with more negative tests should be planned to exclude their impact on sensor performance.

## Figures and Tables

**Table 1:** The values of oxidation potential (E_OX_), reduction potential (E_RED_), separation peak (ΔE) for the [Fe(CN)_6_]^3-/4-^ redox system and electrolyte resistance (R_e_), constant phase element (CPE), the parameter of constant phase element, exponent (n), and charge transfer resistance (R_ct_) calculated from the EEQC model.

SAMPLE	E_OX_/mV	E_RED_/mV	ΔE/mV	Re/Ω	CPE/µFΩ^−1^s^n^	n	R_ct_/Ω
bare Au	202	108	94	148	41.4	0.492	190
Au/4-ATP	201	98	103	153	4.1	0.704	256
Au/4-ATP/anti-Spy	237	72	165	161	2.9	0.715	446
Au/4-ATP/anti-Spy/BSA	285	52	233	191	1.01	0.862	1790

**Table 2:** The comparison of *S. pyogenes* detection methods.

Method	Target Analyte	Linearity Range	LOD	Year	Ref
piezoelectric	bacterial cell	3 × 10^2^–3 × 10^6^ cfu/mL	12 cfu/mL	2014	27
DPV	ssG-DNA	10^−3^–10^−1^ ng/6 μL	130 fg/6 μL	2017	29
DPV	ssG-DNA	0–1 ng/6 μL	0.01 ng	2014	30
CV	ssG-DNA	0.5–50 ng/6 μL	0.01 ng/6 µL	2017	33
CV	ssG-DNA	0–7.5 ng/6 µL	0.10 ng/6 µL	2016	35
EIS	bacterial cell	100–10^5^ cells/10 μL	100 cells/10 μl	2013	37
EIS	bacterial cell	4.2 × 10^2^–4.2 × 10^6^ cfu/mL	9.3 cfu/mL	2020	This work
